# Assessing predictors of students’ academic performance in Ethiopian new medical schools: a concurrent mixed-method study

**DOI:** 10.1186/s12909-023-04372-4

**Published:** 2023-06-17

**Authors:** Hafte Teklay Gebru, Daniëlle Verstegen

**Affiliations:** 1 Department of Biomedical Sciences, College of Health Sciences, Aksum University, P.O.Box: 298, Axum, Ethiopia; 2 School of Health Professions Education, Maastricht University, Maastricht, the Netherlands

**Keywords:** Academic Performance, Medical education, NMEI

## Abstract

**Background:**

Since 2012 the Ethiopian Federal Ministry of Health and Education implemented a new medical curriculum in 13 institutions. The new curriculum introduced some questions on its admission policy: students can join with different educational backgrounds. Students’ performance on qualifying exams and grade point average are lower than desired. Therefore, the aim of the study was to investigate what factors predict the academic performance of students in the New Medical Education Initiative in Ethiopia.

**Methods:**

A concurrent mixed method of survey and qualitative was used; for the survey, a structured self-administered questionnaire was distributed to students of four randomly selected medical schools from December 2018 to January 2019. The questionnaire includes questions about socio-demographic and educational background of participants. Multiple linear regression analysis was used in order to identify the factors associated with academic performance. In-depth interviews were conducted with 15 key informants to explore qualitatively.

**Results:**

In the multiple linear regressions, stress was associated with lower academic performance. Students with prior education in the field of health science outperformed students with other bachelors. The cumulative grade point average of the previous bachelor degree and the score on the entrance exam to join medicine also significantly predicted performance. Although some more variables are identified from the qualitative interviews, its findings supported the survey results.

**Conclusions:**

Of the number of predictor variables analyzed in the model, only stress, prior educational degree, performance in the prior degree and entrance exam score were significantly correlated with the performance of students in their preclinical medical engagement.

## Background

Medical education is in a continuous evolution with regards to its aim and structure. For the last fifty years, changes have been primarily aimed at maximizing competencies through providing contextualized, problem-oriented and self-directed learning to produce more competent medical professionals [1–3]. In Ethiopia medical education was traditional i.e. lecture-based and graduates were missing some competencies like communication skills [4]. In such a lecture-based curriculum, students are passively engaged in the educational process and courses are discrete and discipline based [5]. Since 2012, the Ethiopian Federal Ministry of Education in collaboration with the Ministry of Health has implemented a new curriculum, the New Medical Education Initiative (NMEI) in 13 medical schools which is characterized by Problem-Based Learning (PBL) [6]. Empirical evidence has shown that, PBL stimulates communication skills, deep learning and better development of competencies [7]. In this system, integrating basic sciences to clinical courses earlier and different types of educational methods are employed [7, 8]. That is why NMEI has changed over to a PBL curriculum.

Academic performance (AP) of students is affected by different factors; socio-demographic factors like, financial income and gender were found to be affecting academic performances [9–12]. Some factors like work experience have a positive effect on academic performance and others, like stress, were found to be negatively affecting students’ performance [13, 14].﻿ Student performance is correlated with different factors related to prior education, such as entrance qualification, level of prior education and prior achievement [9, 15, 16]. The presence of experienced teachers with higher degrees and technology support, like internet access, enhance student performance [17, 18]. A study from University of Michigan confirmed that competent, knowledgeable teachers contributed a lot to improved achievements [19]. Staff development includes strategies which ensure academicians are equipped with new and advanced knowledge and skills to maximize medical education [20]. Continuous skills training to teachers resulted in students’ improved preparation and imposed positive influence on their learning during their clinical study [21]. In comparison cohort studies, students of PBL curricula outperform students of lecture-based curriculum in exams of deeper understanding and cognitive skills related to patient management [22]. PBL students had better learning outcomes; they showed deeper learning approaches and better academic performances [23–25]. However, there are also studies which reported that there is no significant difference in the performance of the students from the two systems [26].

From the literature, many factors can affect student performances with varying impact and not all these factors are examined in this study. The focus of this study will be on socio-demographic factors and prior education related factors. These factors seem to play an important role on AP of students, but research has mostly been done in Western high-income countries. We do not know much about this in non-Western, low-income like Ethiopia and there are some differences in the target group of students. Important contributing factors of success should be promoted and impeding factors should be tackled [10] to maximize students’ performance because poor academic success and failure rates result in higher attrition rates, reduced number of graduates and increased educational cost. Hence, AP becomes the business of educational researchers even in low-income countries like ours.

Even though, NMEI utilizes modern educational principles, students are poorly performing in the qualifying exams and in their cumulative grade point average (CGPA). It is not clear why and we do not know which variables can predict students’ achievements. Evaluations (SWOT analysis) indicate that there might be a problem with the admission policy. Some stakeholders argue that integration of basic science into clinical courses would be easier for those who have a background in health professions (bachelor and work experience) than for those who have a bachelor from another domain [27]. Hence, the study was aimed at assessing socio-demographic and education related factors that predict the academic performance of students in the NMEI schools of Ethiopia and the following question was addressed by this study: which socio-demographic and education related factors predict the academic performance of preclinical medical students?

## Method

### Study design

A concurrent mixed method design was employed; an institution based survey with additional explorative interviews. A survey was used to gather information from a larger group of participants. Interviews were used to gain more insight in how different factors may influence Academic Performance.

### Study context

There are 13 medical schools in Ethiopia with the NMEI track and all the schools are graduate-entry programs. Applicants with a first degree are considered for admission to NMEI schools. Students are enrolled from various health and natural science backgrounds. They need to pass the entry exam. Many students have prior work experiences with paid salary and some of them also have their own family. In NMEI schools the study duration is four and half years. Students study the normal and abnormal function and structure of human body for the first two years, followed by two clinical years and the final half a year is block internship time.

The main feature of NMEI program is integrating basic sciences to clinical skills complemented by early clinical attachment and frequent community exposure, the use of PBL and professional competency development (PCD) all of which need active engagement of the students. The curriculum utilizes a modular system and provides horizontal and vertical integration of basic and clinical subjects. During the first two years of study, students focus on PBL, PCD and community attachment sessions; assisted by lectures, skills training, basic science laboratory and clinical attachments. Social and population health courses are longitudinally given throughout the students’ study.

### Participants

All students of medical schools with the new PBL-based curriculum in Ethiopia were the source population. Of the 13 NMIE schools in the country, four schools (30%) were selected randomly by lottery method. More than 890 students are enrolled in the schools. The selected schools were Adama Hospital medical college, Axum University medical school, Yekatit-12 Hospital medical college and Debre-Markos University medical school. From selected schools, all students whose CGPA was available were included in the study. Internship students may be challenged to answer the questionnaire and to remember their preclinical situation retrospectively. In addition, they are assigned to different hospitals which made it difficult to access them for data collection. Therefore, they were excluded from the study. There were a total of 397 students in the selected schools but those who met the inclusion criteria were 312. For the qualitative part, a face to face in-depth interview was conducted with 15 key informants.

### Instruments

A structured self-administered questionnaire prepared in English was distributed among the students. The questionnaire has been developed from relevant literature including measurement for the stress by a standardized tool called Medical Student Stressor Questionnaire (MSSQ) [28, 29].

The questionnaire has 49 items and consists of;

- ▪ Basic items used to collect socio-demographic characteristics of the participants with 6 multiple choice items and 4 open questions.
- ▪ The MSSQ has 34 items on a 5-point Likert–scale, including the following scales; Academic related stressors (13 items), Intra & interpersonal related stressors (7 items), Teaching and Learning stressors (7 items), Social related stressors (3 items), and Group activities related stressors (4 items).
- ▪ Five items to investigate factors related to prior education (one multiple choice item about prior educational degree and 4 open items about performance in previous degrees and exams).

The MSSQ is a standard and validated questionnaire but the other items were added by the researchers and customized to the educational system in Ethiopia. To ensure data quality, the questionnaire was pilot tested in Wollo University medical school (a school not included in this study) among 23 students. After the pilot test, some editorial improvements were done to increase clarity to the participants. For reliability of the tool a Cronbach’s alpha of 70% was recorded. As to the qualitative part, we designed four semi structured questions focused to identify the challenges students often face in the curriculum, the prominent factors affecting students’ performances and reasoning out why. Finally, participants’ recommendations on how to improve performances in the program were forwarded. During the explorative interviews, common causes of poor performance and the impact of different factors were explored. Interview was done in local language (Amharic) to ensure rich explanation; it was continued until saturation by probing. Training was provided to the interviewers.

There are two possibilities to measure the students’ academic performances; the qualification exams and the cumulative grade point average (CGPA). Qualification exam consists of 60% from the CGPA and 40% end year exam; it is a pass (≥ 60%) and fail (< 60%) assessment and it is provided at the end of second year. CGPA shows the averaged grade point for all the modules covered in the course of two years. Letter grades are given based on points earned out of 100 for each module. It has a fixed scale and linked to the curriculum. The letter grades and points scored out of 100 are listed here; (A: ≥ 85, B^+^: 80–84.99, B: 70–79.99, C^+^: 65–69.99, C: 60–64.99, D: 50–59.99 and F: < 50 respectively for excellent, very good, good, satisfactory, fair, below pass mark and failed performers).

Qualifying exams are not appropriate to measure the AP for two important reasons; first, the CGPA accounts 60% of the qualification assessment score and only 40% is calculated from the end year exam. Second qualifying assessments are given only for some randomly selected modules (commonly 6 modules out of eleven) of the two years’ study. Using it for measuring AP is just double consideration of the CGPA (at least the 60%) as an outcome variable; this causes statistical fallacy. CGPA is the average of the two years’ performance (for all the modules) which provides relatively valid assessment results of the students. It allows richer information about the students’ performance. Hence, CGPA indicates the actual students’ academic performance better than qualification exams where it was converted into 100 points.

### Procedure

Data collection was done among medical students who completed their preclinical years from December 2018 to January 2019. It was done in the classrooms of the respective schools. Five teachers were recruited and oriented about the data collection technique and research purpose. Participants were first oriented about the objective of the study, contents of the questionnaire and its organization. Then, the questionnaire was distributed among the students on paper and filled in immediately. To explore the factors affecting the students’ academic achievement, a face to face in-depth interview was conducted withg 15 key informants who were selected purposefully (6 students and 9 teachers). Interviews lasted for a maximum of 26 min. Two experienced and trained interviewers took audio records and written notes during the interview time. Data collection continued until saturation.

### Data analysis

Collected data was reviewed and checked for completeness and twenty incomplete questionnaires were discarded. Collected data was entered into Epi-info version 7.0 software package and analysis was performed using SPSS version 22. For reporting descriptive statistics were collected and presented in tables, graphs and text. The statistical analysis was made at 95% confidence interval. ANOVA was tested to compute the model significance and it was found to be a good fit (F-test = 6.56 at significance level of *P* < 0.001). The statistical model can explain 71% of the variation (R squared value = 0.71 with *P*-value < 0.001). The relationship of dependent and independent variables was determined by employing multiple linear regression analysis after checking for assumptions like normal distribution, independence of individual observations and linear relationship of the Academic Performance (AP) with each independent variable. The multi-collinearity was checked by using tolerance and variable inflation factor (VIF) tests and a *P*-value less than 0.05 was used to check statistical significance. In the qualitative part, an experienced and qualified transcriber was employed. The recordings and written notes were transcribed and after repeated reading they were coded manually into themes, using an inductive approach. Quotes are presented with the quantitative findings, where relevant.

## Results

### Socio-demographic characteristics of the participants

A total of 312 students were approached in the study with 93.58% response rate (*n* = 292). The mean age of participants was about 28.50 years. The average monthly income was 30 USD. The students had an average working experience of 2.97 years before they started to study Medicine. The other socio-demographic detail of the study participants is presented below (Table 1).

**Table 1:** Socio-demographic characteristics of the study participants (*n* = 292)

Characteristic	Category	*Number*	Percent
Gender	Males	268	91.8
	Females	24	8.21
Residence	Campus dormitory	277	94.9
	Non-dormitory	15	5.1
Marital status	Single	234	80.1
	Married	58	19.9
Age in years	Mean ± SD	28.48 ± 3.12	
Living with...	Alone	30	10.3
	With Friends	255	87.3
	With Family	7	2.4
Work experience in years	Mean ± SD	2.98 ± 1.254	
Average income per month (USD)	Mean ± SD	30 ± 3.32	
Stress status	Stressed	128	43.8
	Non-stressed	164	56.2
Current level of study	Clinical year one (C-I)	114	39.0
	Clinical year two (C-II)	178	61.0

### Education-related variables of the participants

**Fig. 1:**
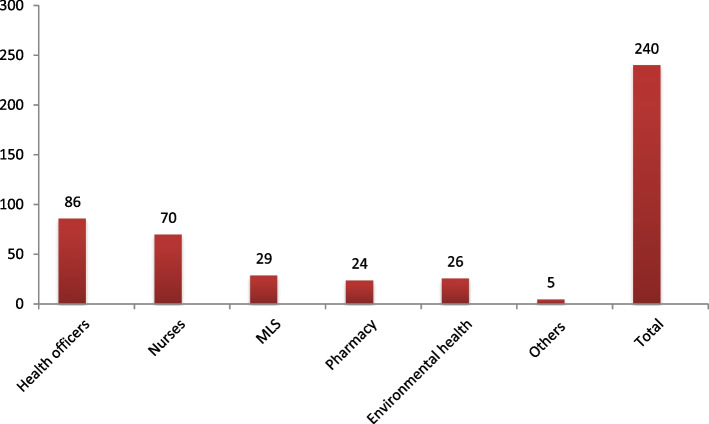
The frequency of study participants for those who had health science educational background with regard to the type of their prior degree, (*n* = 240)

Most of the students’ prior educational background is in the domain of health sciences: (82.20%) followed by Biology and Chemistry which account for 7.50% and 3.80% respectively. The students’ mean CGPA for their bachelor degree (i.e. academic achievement in their prior studies) was 3.42 on average with a minimum of 2.10 and maximum of 4.00. The average score of students (out of 100) for the entry exam to join medicine was 58.20 ± 5.75. In the preclinical qualifying exam, students’ average performance was 75.10** ± **6.67. The average academic performance of students was computed in percentages; it became 78.55% ± 7.01. Further detail is provided in the table below (Table 2). When we compute the frequencies for the students with their prior education in the health sciences, most (35.80%) of them were health officers followed by nurses (29.20%). There were comparable number of pharmacists and Environmental health professionals (Fig. 1).

**Table 2:** Education related characteristics of the study participants (*n* = 292)

Characteristic	Category	*Number*	Percent
Prior degree students to join NMEI	Health Sciences	240	82.20
	Non-health sciences	52	17.80
CGPA of students’ prior education	Mean ± SD	3.42 ± 0.25	
Entrance exam score	Mean ± SD	58.20 ± 5.75	
Pre-clinical qualifying exam score	Mean ± SD	75.10 ± 6.67	
Academic Performance (AP)	Mean ± SD	78.55% ± 7.01

From the correlation matrix it is clear that age had weak negative relationship with students’ academic performance (Pearson’s Correlation = 0.030 with *P* value of 0.61) and it was not statistically significant. However, as indicated in the table prior performance (Pearson’s Correlation = 0.67 with *P* value of 0.012) and entrance exam score to join medicine (Pearson’s Correlation = 0.79 with *P* value of 0.001) had significant positive relationship with academic performance (Table 3).

**Table 3:** Correlation matrix for the predictors of the students’ academic performance (*n* = 292)

Independent Variable	Pearson’s correlation with AP	*P*-value
Age	-0.03	0.61
Work experience	-0.10	-0.08
Average income per month (USD)	0.42	0.47
Performance in prior degree	0.67	0.012
Entrance exam score	0.79	0.001
Family size	0.02	0.71

### Predictors of academic performance of students

All the pre-specified variables met the criteria to be selected for multiple linear regression model of analysis. The collinearity test was performed using tolerance and variable inflation factor (VIF). The collinearity statistics showed that both the marital status and family size were at VIF of 24.994 and 25.277 respectively which indicates they have multi-collinearity effect. Therefore, family size was removed from the model. The multi-collinearity for the rest variables showed a tolerance near to one and VIF was < 2.

Among the thirteen variables entered and run for multiple linear regression, four were found to have statistically significant effect on the students’ academic performance. Stress, the type of prior educational degree, the performance during the prior degree measured in CGPA and the score of the entrance exam for Medicine significantly affected performance (that is the *P*-value was < 0.05). The regression model can significantly explain the established relationships between the independent variables and the academic performance. In the multiple linear regression, students with stress perform worse than students with no stress; the academic performance was reduced by 2.22% (Adjusted B = -2.218, 95% CI = -3.734–0.702). A preclinical coordinator stated that *“Since the curriculum is tight and intensive, students are often tensioned to study all the topics in a certain module”* and one graduated doctor said that *“the parallel modules from public health (social and population health courses) impose extra study load which made my stay very tough and difficult”*.

When their prior education was in non-health sciences, students’ performance was lowered by 6.10% (Adjusted B = -6.089, 95% CI = -8.103, -4.076). One second year student said that, *“In different learning engagements and exams, I found myself more poorly performing than most of the students. For example; students from health science area talk more about ideas in PBL sessions but I contribute less to the PBL because I am from mathematics”.*

With regard to the prior performance of the students, when the cumulative grade point average (in prior education) is increased by one, the academic performance of the students would be increased by almost 6% (Adjusted B = 5.976, 95% CI = 2.909, 9.044). *“Medicine is primarily just about reading, but the prior performance and profession may still persist with those who are better performers in their prior degree, however, this is not always true; we know many students who were outstanding in their prior degree but who are dismissed in this program”*(a third year student). As the students’ entrance exam is increased by one, performance of students improved by 0.2% (Adjusted B = 0.201, 95% CI = 0.067, 0.336) (Table 4).

**Table 4:** Multiple linear regression analysis of the predictors of the students’ academic performance (*n* = 292)

Variables	95.0% Confidence Interval for B		Adjusted *P*-value
	**Crude beta (CI 95%)**	**Adjusted beta (CI 95%)**	
Age	-0.068 (-0.328, 0.192)	1.382(.218, 0.875)	0.799
Residence			
Campus dormitory	0	0	
Non-dormitory	2.240 (-1.416, 5.897)	1.834(-1.676, 5.34)	0.326
Living with...			
Alone	0	0	
With friends	0.897 (0.663, 0.508)	0.707(-1.747, 3.161)	0.571
Marital status			
Single	0	0	
Married	-0.408 (-0.2.436, 1.619)	0.088 (-1.888, 2.065)	0.930
Income	0.000(0.000, 0.001)	-2.254 (0.000,0.000)	0.993
Gender			
Males	0	0	
Females	-0.518(-3.464, 2.427)	1.382 (-1.383, 4.15)	0.326
Stress			
Non-Stressed	0	0	
Stressed	-2.653 (-4.255, -1.051)	-2.218 (-3.734, -0.70)	**0.004**
Working experience	-0.568 (-1.211, 0.075)	-0.011 (-0.70, 0.665)	0.976
Prior educational degree			
Health	0	0	
Non-health	-6.248 (-8.236, -4.260)	-6.089 (-8.103, -4.1)	** < 0.001**
Prior performance in CGPA	4.102 (0.914, 7.291)	5.976 (2.909, 9.044)	** < 0.001**
Entrance exam score	0.240 (0.102, 0.378)	0.201 (0.067,0.336)	**0.004**

## Discussion

In this study, we found that stress, prior educational degree, performance in the prior educational degree and the entrance exam to join medicine were related to the academic performance of NMEI preclinical students.

The proportion of students who were stressed in the current study was 43.8%. which is lower compared to reports from studies conducted in Pakistan (> 90%), India (73%), Thailand (61.4%) and Jimma (52.4%) medical schools [30–33]. The difference may have occurred because the participants in the current study were older compared to the participants in other studies so that they may have better coping strategy to stress.

However stress affected the performance of the students in the Pakistani students [31, 34] and also in the current research participants. A research from Jimma University medical school, reported that stress had negative correlation with academic performance (R = -0.273, *P* = 0.001) [33]. Stress definitely causes negative impact on academic performances, physical and mental health and long term learning and employment attainment but enhancing stress coping strategies and/or improving personal stress management skills can play a significant role to reduce the effect of stress [35, 36].From the interview, participants supported this finding, in low-income countries economic strain can be a cause of stress, especially because in this program students might be married and responsible for a family with children by the time they study Medicine. In addition, the study schedules are tight and stressful. Stress is characterized by mental or emotional state of strain which reduces students’ concentration, self-esteem and may cause anxiety and depression which would have negative impact on academic performance [34, 37]. This will be high if academic and personal counseling service is less practiced.

The prior education of the students also matters: students with a background in the health science sector have been exposed to patient care and human biology and that is probably why they outperform the students with a background that is not in the health sciences. When we examine the impact of the prior education on the performances, it seems higher but there are no specific prior studies to compare. We expect that this effect is not only due to the entry level of knowledge about the basic sciences, but also due to the fact that these students have engaged in clinical visits, community education, and social and population health sciences. From the qualitative finding, most of the key informants supported this idea by pointing out that, educational background matters. Moreover, the curriculum is integrated (between preclinical and clinical courses) so that students with health prior education can use their prior knowledge during assessments so that they score better results.

As to the prior performance of the students, the AP in the medical program was increased by almost 6% up on increasing the prior performance by 1% and a research on predictors of AP in the discipline-specific bioscience paper conducted at Auckland University among oral health students revealed that prior academic achievement was a reliable predictor of academic performance. For every unit grade increase in prior achievement, there was an increase by 0.812 in the outcome variable (discipline-specific bioscience paper) [9]. This is lower compared to the current report which may be related to the expertise or the level of the students’ prior education. The persistent trend of high grade inflation in Ethiopian schools may also be another reason [38]. Although prior knowledge and performance may relate to the lifelong academic achievement, it cannot be a guarantee because Medicine is all about reading and updating oneself.

In this report, performance in the entrance exam contributed 0.2% increase in the AP and another study from Ethiopia revealed that entrance exam has strong correlation with academic performance. Students who scored 50s will be treated under the same academic system with those who scored 80s in the current study. This may have positive or negative impact on the students’ performance [38]. Various studies from different parts of the world reported that prior performance is directly correlated to the actual academic performance of students [15, 39]. The academic achievement of college students was found to increase by 0.53% for every increase in the prior performance [15] which is higher than the current report. This difference may be due to the nature and behavior of the participants as the prior study was conducted among accounting students.

Using the linear regression avoids missing of specific dataset when categorization of measurements and logistic model is employed. The effect of confounders was controlled to minimum and reliability test was also checked and was found to be appropriate.

Sharing this finding to different stakeholders who have potential influence on curriculum change and revision would assist them to improve the admission policy. The schools are advised to work more on student mentorship to support students to reduce stress and its negative effect. Advisors or mentors should support students in designing effective strategy to manage stress. There is few or no existing educational researches in our country, so the current study can provide a baseline source of educational study for other researchers. It is better to measure all years AP in a holistic approach to rich at comprehensive conclusions.

### Limitations of the study

The current study addressed only two groups of explanatory variables, the socio-demographic and educational; which can explain limited variations of the outcome variable (AP). That is why other variables are identified in the interview. This research is also limited to performance of preclinical students. However, assessing all performances including clinical one would be very important to see the whole academic performance in a conclusive way. Measuring clinical performance is as important as the preclinical one because it provides comprehensive understanding of the determinant factors for students’ Academic Performance. We have the lack of empirical evidences regarding educational researches mainly in countries with similar educational set-up.

## Conclusion

From the examined variables only stress, Prior educational degree, Prior performance in CGPA and Entrance exam score to join Medicine statistically and significantly predicted the performance of the students. These findings will help improving the selection procedures and designing measures to support students in coping with stress. Most of the quantitative findings were supported by the opinions of the key informants.

## Data Availability

The data and other materials are available from the corresponding author.

## Abbreviations

AP: Academic Performance
CGPA: Cumulative Grade Point Average
CI: Confidence Interval
NMIE: New Medical Education Initiative
PBL: Problem Based Learning
PCD: Professional Competency Development
